# Supplementary material for: Ankyrin-R regulates fast-spiking interneuron excitability through perineuronal nets and Kv3.1b K+ channels
Source: eLife. 2021 Jun 28;10:e66491. doi: 10.7554/eLife.66491 (PMC8257253; doi:10.7554/eLife.66491)
Supplement: Supplementary file 1. — Data are from 3 Ank1F/F, 2 Ank1+/+;Dlx5/6-Cre, and 4 Ank1F/F;Dlx5/6-Cre mice, and are reported as mean ± SEM (number of cells). Bolded p values indicate significance. [file elife-66491-supp1.docx]

| **Supplementary File 1.** |  |  |  | **p value (One-Way ANOVA or Kruskal-Wallis test)** | | |
| --- | --- | --- | --- | --- | --- | --- |
|  | ***Ank1^F/F^*** | ***Ank1^+/+^; Dlx5/6-Cre*** | ***Ank1 ^F/F^; Dlx5/6-Cre*** | ***Ank1 ^F/F^* vs. *Ank1^+/+^; Dlx5/6-Cre*** | ***Ank1 ^F/F^* vs. *Ank1^F/F^; Dlx5/6-Cre*** | ***Ank1^+/+^; Dlx5/6-Cre* vs. *Ank1^F/F^; Dlx5/6-Cre*** |
| Resting membrane potential (mV) | -66.69 ± 1.373 (19) | -70.42 ± 0.934 (17) | -68.8 ±  1.102 (30) | 0.1176 | 0.403 | 0.6037 |
| Input resistance (MΩ) | 109 ±  10.56 (19) | 131.7 ± 15.94 (17) | 129.6 ±  7.544 (30) | 0.6384 | 0.1419 | >0.9999 |
| Membrane capacitance (pF) | 82.27 ± 4.849 (19) | 81.85 ± 25.74 (17) | 67.17 ±  4.068 (30) | 0.9983 | 0.0708 | 0.0956 |
| Rheobase current (pA) | 307.1 ± 24.56 (19) | 315.9 ± 26.85 (17) | 300.5 ±  17.75 (30) | 0.9652 | 0.9743 | 0.8768 |
| Action potential latency (ms) | 128 ±  39.41 (19) | 152.2 ± 44.77 (17) | 43.71 ±  18.01 (30) | >0.9999 | 0.0973 | ***0.0246*** |
| Action potential threshold (mV) | -33.51 ± 1.107 (19) | -33.09 ± 1.416 (17) | -37.62 ±  1.087 (30) | 0.9766 | ***0.0165*** | ***0.0116*** |
| Action potential amplitude (mV) | 50.88 ± 2.419 (19) | 53.12 ± 2.858 (17) | 50.41 ±  1.965 (30) | 0.8152 | 0.9881 | 0.6962 |
| Action potential half-width (ms) | 0.3765 ± 0.015 (19) | 0.3486 ± 0.014 (17) | 0.5316 ±  0.001 (30) | 0.3124 | ***<0.0001*** | ***<0.0001*** |
| Afterhyperpolarization amplitude (mV) | 26.07 ± 0.773 (19) | 26.9 ± 0.923 (17) | 19.19 ±  0.747 (30) | >0.9999 | ***<0.0001*** | ***<0.0001*** |
| Afterhyperpolarization time (ms) | 1.442 ± 0.119 (19) | 1.318 ± 0.087 (17) | 1.913 ±  0.088 (30) | 0.9943 | ***0.0002*** | ***<0.0001*** |
| Spike frequency adaptation | 0.897 ± 0.025 (13) | 0.9326 ± 0.017 (13) | 0.8912 ±  0.022 (24) | 0.9982 | >0.9999 | 0.721 |
| Spike amplitude adaptation | 0.8243 ±  0.012 (13) | 0.8446 ±  0.075 (13) | 0.6731 ±  0.027 (24) | >0.9999 | **0.0013** | **0.0001** |
| Firing frequency (Hz) | 221.5 ± 9.257 (13) | 329.2 ± 11.06 (13) | 204.6 ±  5.516 (24) | 0.5423 | 0.6966 | ***0.0196*** |
| Depolarization block current (pA) | 1438 ±  12.50 (8) | 1335 ±  59.48 (13) | 1006 ±  77.44 (16) | >0.9999 | ***0.0031*** | ***0.0048*** |
| Maximum dV_m_/dt (V/s) | 235.1 ± 18.24 (19) | 244.8 ± 15.43 (16) | 185.4 ± 8.77 (30) | 0.8899 | ***0.0217*** | ***0.0081*** |

**Supplementary File 1.** Intrinsic properties of WFA^+^ cells in *Ank1^F/F^*, *Ank1^+/+^;Dlx5/6-Cre*, and *Ank1^F/F^;Dlx5/6-Cre* mice. Data are from 3 *Ank1^F/F^*, 2 *Ank1^+/+^;Dlx5/6-Cre*, and 4 *Ank1^F/F^;Dlx5/6-Cre* mice, and are reported as mean ± SEM (number of cells). Bolded p values indicate significance.
