# Supplementary material for: Ankyrin-R regulates fast-spiking interneuron excitability through perineuronal nets and Kv3.1b K+ channels
Source: eLife. 2021 Jun 28;10:e66491. doi: 10.7554/eLife.66491 (PMC8257253; doi:10.7554/eLife.66491)
Supplement: Supplementary file 2. [file elife-66491-supp2.docx]

| **Supplementary File 2**  **Protein and Gene Abbreviations** | | |  |
| --- | --- | --- | --- |
| **Acronym** | **Name** | **Gene** | |
| AnkR | Ankyrin-R | *ANK1* | |
| AnkB | Ankyrin-B | *ANK2* | |
| AnkG | Ankyrin-G | *ANK3* | |
| Pv | Parvalbumin | *PVALB* | |
| SST | Somatostatin | *SST* | |
| TnC | TenascinC | *TNC* | |
| TnR | TenascinR | *TNR* | |
| Vcan | Versican | *VCAN* | |
| Bcan | Brevican | *BCAN* | |
| Acan | Aggrecan | *ACAN* | |
| Nrp1 | Neuropilin-1 | *NRP1* | |
| WFA | *Wisteria Floribunda* | |  |
|  |  | |  |
| **Structural Abbreviations** | | |  |
| **Acronym** | **Name** | |  |
| CAM | cell adhesion molecule | |  |
| PNN | perineuronal net | |  |
| AIS | axon initial segment | |  |
| ECM | extracellular matrix | |  |
| CNS | central nervous system | |  |
|  |  | |  |
| **Technical Abbreviations** | | |  |
| **Acronym** | **Name** | |  |
| IP | immunoprecipitation | |  |
| PSM | peptide spectral match | |  |
| WT | wildtype | |  |
| KO | knockout | |  |
| UA | unit area | |  |
| AHP | afterhyperpolarization | |  |
| mEPSC | miniature excitatory postsynaptic currents | |  |
| mIPSC | miniature inhibitory postsynaptic currents | |  |

**Supplementary File 2.** Abbreviations used throughout the paper.
